# Titanium Dioxide Nano-Formulation: Characterization, Antimicrobial Activity, and Wound Healing in Animals

**DOI:** 10.3390/ani13172688

**Published:** 2023-08-22

**Authors:** Noppason Pangprasit, Yada Thammawong, Alongkorn Kulsirorat, Phongsakorn Chuammitri, Aphisek Kongkaew, Montira Intanon, Witaya Suriyasathaporn, Surachai Pikulkaew, Wasana Chaisri

**Affiliations:** 1 PhD’s Degree Program, Department of Food Animal Clinic, Faculty of Veterinary Medicine, Chiang Mai University, Chiang Mai 50100, Thailand; panny.2.yaa@gmail.com; 2 Department of Food Animal Clinic, Faculty of Veterinary Medicine, Chiang Mai University, Chiang Mai 50100, Thailand; yada.thammawong@gmail.com (Y.T.); a.kulsirorat@gmail.com (A.K.); suriyasathaporn@hotmail.com (W.S.); surachai.pikul@cmu.ac.th (S.P.); 3 Department of Veterinary Bioscience and Veterinary Public Health, Faculty of Veterinary Medicine, Chiang Mai University, Chiang Mai 50100, Thailand; phongsakorn.c@cmu.ac.th (P.C.); montiraintanon@gmail.com (M.I.); 4 Research Center of Producing and Development of Products and Innovations for Animal Health, Chiang Mai University, Chiang Mai 50100, Thailand; 5 Research Administration Section, Faculty of Medicine, Chiang Mai University, Chiang Mai 50200, Thailand; moromo046@gmail.com

**Keywords:** animal wound, anti-biofilm-forming, antimicrobial activities, skin microflora, titanium dioxide

## Abstract

**Simple Summary:**

In veterinary fields, infected wounds are one of the leading causes of morbidity and mortality in animals. Due to the widespread use of antibiotics in wound treatment, multidrug-resistant bacterial strains have emerged in animals and interest in alternative antimicrobial treatments has focused on the use of metal oxide nanoparticles. In this study, we have addressed this challenge using the application of a solution-stabilized dispersion of titanium dioxide nanoparticles (TiO_2_-NP). This product has been investigated for its unique properties of countering specific members of the skin microflora and its enhancement of the wound healing process. It has been shown that TiO_2_-NP with 383.10 ± 23.05 nm and the zeta potential 19.27 ± 4.65 mV are able to inhibit bacterial growth and biofilm formation of reference members of the skin microflora, *Staphylococcus aureus* and *Escherichia coli*, and enhance cell migration and wound healing processes in vitro and in vivo. TiO_2_-NP is a promising candidate for veterinary medical applications that can be used to improve the wound healing process.

**Abstract:**

The use of metal oxide nanoparticles as an alternative antimicrobial agent has gained attention due to the increasing problem of antimicrobial resistance. Understanding its properties and potential benefits can contribute to the development of more effective and sustainable treatments in veterinary medicine. The aim of this study was to characterize TiO_2_-NP formulations and evaluate their antibacterial and wound healing abilities. The diameters and zeta potentials were determined using the Zetasizer in conjunction with dynamic light scattering. The agar-well diffusion method, time-kill kinetic assay and crystal violet assay were used to evaluate their antimicrobial activities. Wound healing assays were conducted both in vitro and in vivo. The study demonstrated that TiO_2_-NP formulations exhibit significant antimicrobial properties against various bacterial strains such as *S. aureus* and *E. coli*. No measurable *E. coli* growth was observed within a 15-min period following exposure to TiO_2_-NP formulations. The TiO_2_-NP formation can improve wound healing by enhancing cell migration and collagen formation in both in vitro and in vivo conditions. In summary, our study suggests that TiO_2_-NP has the potential for use as an antimicrobial agent for animal wound treatment due to its ability to suppress bacterial growth and biofilm formation, as well as to enhance wound healing.

## 1. Introduction

Bacterial infections in animal wounds are a major concern for the global animal healthcare industry. Aerobic or facultative bacterial pathogens such as *Staphylococcus aureus*, *Streptococcus pyogenes*, *Pseudomonas* spp., *Escherichia coli*, *Enterococci* spp., etc. are the most common causative organisms associated with infected wounds [1,2]. When the progression of the wound healing process is disorganized and delayed, it leads to the formation of a chronic wound [3,4]. This adds to the burden by necessitating extended wound care. Generally, broad spectrum antibiotics and antiseptics are frequently used in wound management due to their cost-effectiveness and efficacy [5,6]. The extensive use of broad-spectrum antibiotics is a major contributor to the emergence of resistant bacteria and multidrug-resistant bacteria (MDR) on wound sites [7,8]. Furthermore, they are regarded as reservoirs for the transmission of resistant bacteria to humans through close contact [9,10]. Biofilms, which are formed of extracellular polymeric substances that act as a protective layer, have become a major concern, resulting in chronic wounds, an increase in MDR bacterial strains, and a reduction in treatment efficiency [11]. The antibiofilm properties of various metal oxide nanoparticles as well as their effect on plankton bacteria cells and biofilms [12], including ZnO (500 µg/mL) and TiO_2_ (100 µg/mL) nanoparticles have been demonstrated against strong and weak biofilm-producing methicillin-resistant *Staphylococcus aureus* (MRSA) isolates [13].

Nanotechnology is a recognized and reliable research field for improving the efficacy of treatment. Metallic or metal oxide nanoparticles such as silver, gold, zinc and titanium have emerged as the most promising and rapidly emerging materials for alternative treatment in wound healing therapies in order to increase efficacy or improve clinical outcome and reduce the likelihood of the emergence of multidrug-resistant and biofilm-forming bacteria [14,15].

Among the various metal oxides, the titanium dioxide nanoparticle (TiO_2_-NP), a photoactive metallic nanoparticle, has recently shown promise in modern biomedical applications [16]. TiO_2_-NP has distinct and unique properties such as an electrical and photocatalytic effect and has a wide range of applications [17]. Compared to other antimicrobial agents, TiO_2_ in nanoparticle size has received considerable attention due to its stability, safety, and broad-spectrum antibacterial effectiveness as well as being friendly to the environment [18]. Thus, a TiO_2_-NP formulation with these properties is expected to create favorable consequences for therapeutic treatment of wounds. Although there are now some reviews exploring the antimicrobial efficacy or wound healing properties of the TiO_2_-NP formulation [17,19], only a few reviews have been published on the topic of the TiO_2_-NP formulation specifically used to improve the healing of infected wounds in animals. Therefore, the purpose of the present study is to provide information about the characterization, antimicrobial, anti-biofilm-forming, and enhanced wound-healing properties of the TiO_2_-NP formulation for the treatment of infected wounds in vitro and animal models.

## 2. Materials and Methods

### 2.1. Preparation and Characterization

The TiO_2_-NP formulation with sizes ranging from 10 to 100 nm was obtained from Apogee International Company Limited (Bangkok, Thailand). The prepared stock solution was sonicated for 5 min before being used in each assay. The morphology of TiO_2_-NP formulation was observed using transmission electron microscopy (TEM, JEOL JEM-2100, Tokyo, Japan). Particle size was measured by dynamic light scattering (DLS) and zeta potential was measured by laser-Doppler anemometry with the Zetasizer apparatus (Ver. 7.11, Malvern, U.K.), respectively.

### 2.2. Antimicrobial Activities

#### 2.2.1. Agar Well Diffusion Methods

The antimicrobial activity test was carried out in triplicate using the agar well diffusion method described previously by Balouiri et al. [20]. The sterilizing technique was used to prepare Muller–Hinton agar as a standard growth medium (MHA, Difco™ & BBL^TM^, U.S.A.). Two specific reference bacterial strains (*Staphylococcus aureus*; *S. aureus* ATCC25923 and *Escherichia coli*; *E. coli* ATCC25922) were suspended in 9 mL of 0.85% normal saline solution (NSS) and uniformly spread on MHA with a sterile cotton swab at a concentration of 10^7^–10^8^ CFU/mL (McFarland density = 0.5). Subsequently, wells of 6 mm diameter were created with a sterile cork borer. Each well was filled with 35 µL of TiO_2_-NP formulation and wells containing the same volume of solvent (35 µL) were used as a negative control, while a standard antibiotic solution of Gentamicin (a final concentration of 10 µg) were used as the positive controls, incubated at 37 °C for 24 h. Antimicrobial activity of TiO_2_-NP formulation was evaluated by observing the appearance of the zone of inhibition and measuring it in millimeters using a Vernier caliper. The growth inhibition data was obtained in triplicate and presented as the average value along with the standard deviation.

#### 2.2.2. Time-Kill Kinetic Assay

A time-kill kinetic assay with some modifications was performed following the method described by Appiah et al. [21]. In brief, the bacterial suspension was adjusted to a concentration of 10^5^–10^6^ CFU/mL in Muller–Hinton broth (MHB, Oxiod, Thermo Scientific™, U.K.) containing the TiO_2_-NP formulation with a concentration equal to the commercial product (Ratio 1:1) and exposed for 0, 5, 15, 30, 60 min and 4, 6, 12 h at 37 °C. The samples were serially diluted 10-fold in phosphate-buffered saline (PBS) and 100 µL of each sample was inoculated aseptically using the spread plate technique onto standard plate count agar (PCA, Oxiod, Thermo Scientific™, U.K.). This method was used to calculate the colony-forming units (CFU) of bacteria in the samples. The plate with the least dilution that contained countable (25–250 CFU/inoculation) colony-forming units was recorded. The average number of colony-forming units in triplicates was calculated as the mean number of colonies.

### 2.3. Antibiofilm-Forming Activity

The crystal violet assay, described by Stepanović et al. [22] with some modifications, was used to quantify biofilm-forming activities. Individual wells of a polystyrene 96-well microtiter plate (flat bottom) were filled with 180 µL of tryptic soy broth (TSB, Himedia™, India) containing 1% glucose and 20 µL of the bacterial suspension (0.5 McFarland scale: 10^7^–10^8^ CFU/mL). In the treatment wells group, 200 µL of TiO_2_-NP formulation was diluted from the stock solution to equal the commercial concentration (ratio 1:1), and then 2-fold diluted in TSB before being incubated at 37 °C for 24 h. After incubation, the content 200 µL of each well were discarded by aspiration and the wells were washed three times with phosphate-buffered saline (PBS, pH 7.2) to remove non-adherent cells and air-dried for 30 to 45 min. Subsequently, 200 µL of absolute methyl alcohol was added for 20 min to fix the adhered cells, and then 200 µL of 0.5% crystal violet were added and the plates were incubated in the dark for 15 min.

After removing the dye solution and washing with sterile distilled water, the attached dye was solubilized with 0.5% (*v*/*v*) ethanol and the optical density of the adherent biofilm was measured using an absorbance of crystal violet at a wavelength of 570 nm in a microtiter plate reader [23]. All assays were performed in triplicate and the standard deviation of the mean was calculated. Based on the resulting average optical density (OD) values, the isolates were categorized as non-producing, weak-producing, moderate-producing, and strong-producing for purposes of interpretation. In comparison to the OD values of the negative control, TSB solution without TiO_2_-NP formulation was used as the negative control (OD_NC_), whereby their OD was subtracted from the experimental strains that were treated with TiO_2_-NP formulation. The biofilm-forming abilities were categorized as shown in Table 1.

### 2.4. In Vitro Wound Healing Assay

#### 2.4.1. Cell Culture

The porcine kidney cells (PK15, ATCC CCL-33) were propagated and maintained in Dulbecco’s Modified Eagle Medium (DMEM) (Gibco, Thermo Fisher Scientific, Waltham, MA, USA) containing 10% (*v*/*v*) fetal bovine serum and specific antibiotics (200 IU/mL Penicillin G sodium, 200 g/mL Streptomycin sulfate). Before being used in wound healing assays, cells were seeded into 24-well cell tissue culture plates and incubated at 37 °C in a humidified 5% CO_2_ incubator for 24 h to achieve complete spreading of cells and an 80% confluent monolayer.

#### 2.4.2. Scratching Wound Healing Assay

A confluent PK15 monolayer in each well was gently scratched with a sterile 200 µL pipette tip to simulate wounding. The wells were then washed with phosphate buffer saline (PBS) to remove any cellular debris. Following washing, each well was filled with a growth medium (DMEM) containing ten or twenty microliters of TiO_2_-NP formulation. As a control, wells containing only the medium were used. The cells were then incubated at 37 °C with 5% CO_2_ for 24 h. Following incubation, the scratched cell layers were visualized and photographed at a low magnification (4×) using an Olympus CK40 inverted microscope equipped with a digital camera (Olympus Corporation, Tokyo, Japan). ImageJ/Fiji^®^ software (NIH, Bethesda, MD, USA) was used to quantify scratch of wound areas.

### 2.5. In Vivo Wound Healing Assay (Animal Model)

#### 2.5.1. Ethical Approval

All experiments were approved by the Institutional Animal Care and Use Committee (Protocol number: 23/2564), Faculty of Medicine, Chiang Mai University, Thailand.

#### 2.5.2. Experimental Animals

Nine six-week-old healthy Mlac: ICR mice (body weight 30–40 g) were included in the experiment. Animals were acclimatized under standard animal laboratory conditions for 7 days prior to the experiment. All animals in this study were anesthetized with Isoflurane (Isoflurane, USP, Baxter™, U.S.A.), which acted as both a sedative and a long-term pain-killer agent. The backs of the mice were shaved, and then sterilized using an alcohol swab. Then, four circular, full-thickness wounds were created aseptically on the dorsal surface of the mice using a sterile 6 mm biopsy punch and scissors. The wound sites of all animals were inoculated with 20 µL of *S. aureus* ATCC 25923 at 10^8^ CFU/mL (McFarland density = 0.5) to generate infection on wound sites. In this model, wound contraction, early wound healing score and tissue histology were evaluated by using a semi-quantitative scoring system. Bacterial data from the wound were collected 24 h after bacterial inoculation. The number of colony-forming units per milliliter (CFU/mL) was investigated by using drop plate techniques. The mean ± standard was calculated and set as the starting point for wound colonization. The animals were randomly divided into three groups and daily received the treatment for 14 days, each consisting of 3 mice: group I, a negative control group, which received 20 µL of 0.85% NSS; group II, a positive control group, which received 20 µL of iodine solution (1% active iodine); and group III, a treatment group, which received 20 µL of TiO_2_-NP formulation. Before application of the solution onto the mice, the wounds were disinfected with 0.85% NSS to clean the wound surface and remove any debris. Early wound healing score (EHS), percentage of wound contraction and bacterial colonization were assessed for all group members at a terminal time point on days 1, 3, 6, 9, and 14 after wound creation.

#### 2.5.3. Early Wound Healing Score (EHS)

The physical appearance of wound healing was monitored by taking digital photographs to determine the early wound healing score (EHS). The EHS system comprises three parameters, including clinical signs of re-epithelialization (CSR), clinical signs of hemostasis (CSH), and clinical signs of inflammation (CSI). The summation of the points for these three parameters generated the EHS. According to Marini et al. [24], the optimal wound healing outcome is characterized by an ESH score of 10 points, whereas the lowest wound healing outcome is represented by a score of 0 points. The descriptions of early wound healing scoring system are shown in Table 2.

#### 2.5.4. Wound Microbial Assay

The modified drop plate technique described by Naghili et al. [25] was used to determine the total amount of bacteria present on the wound sites. Before collecting a sample from each wound site, the wounds were cleaned by scrubbing with 0.85% NSS to remove any debris. Then, the wound surface was swabbed using a sterile cotton swab and transported to a microbiological lab by reinserting the swab into test tubes containing sterile PBS. To obtain a homogeneous suspension, a volume of 100 µL of the sample solution was mixed with 900 µL of sterile PBS. The sample suspension was subsequently subjected to a 10-fold serial dilution to achieve a final concentration range of 10^−1^ to 10^−5^. A volume of 10 µL of each dilution was carefully placed onto the plate count agar (PCA). The plates were incubated at 37 °C for 24 h. After incubation, the colonies were counted and the average number of colonies was determined as the minimum dilution that contained a countable number of colonies (25–250 CFU/inoculation). The results were expressed in CFU/mL.

#### 2.5.5. Wound Contraction

The size of each wound area was measured using Vernier calipers. The percentage of wound contraction was calculated using the following formula.

W_C_ = [(A_0_ − A_t_)/A_t_] × 100

where W_C_ is the percentage of wound contraction, A_0_ is the original wound area, and A_t_ is the area of wound at the time of measurement (days 0, 3, 6, 9, and 14 post wound creation).

#### 2.5.6. Tissue Collection and Histological Analysis

The skin samples from each group were taken on days 0 and 14 after wound creation from the periphery of the wound, along with normal skin and fragments of infected skin. Samples were fixed in 10% buffered formalin, processed by dehydration and embedded in paraffin blocks. Then, samples in a paraffin block were sectioned into 3 µm-thick sections and stained with hematoxylin and eosin (H&E) stains. Wound healing scores and histological changes in each sample were photographed and observed under a light microscope. The study used a semi-quantitative scoring system based on an ordinal scale with four histological parameters: granulation tissue amount, inflammatory infiltrate, collagen fiber orientation, and collagen pattern. The scoring system was previously established by Sultana and Santos [26,27] and is presented in Table 3. The overall healing score for each group was calculated by summing the individual criterion scores. Lower scores indicated poor healing status (8–11), while middle (12–15) and good (16–19) scores indicated intermediate and good healing status, respectively.

### 2.6. Statistical Analysis

Statistical analysis was conducted using ANOVA and Kruskal–Wallis tests for parametric and non-parametric data, respectively. The purpose was to assess differences between groups on each day, including wound contraction rate, bacterial concentration, early wound healing score, and histopathology parameter score. Post-hoc multiple comparison tests were conducted to identify significant differences between groups with a significance level of 0.05, following ANOVA and Kruskal–Wallis tests. The paired *t*-test was used to compare the starting day (Day 0) with the treatment days (Day 3, 6, 9, and 14) following surgical procedures. Violin plots were generated using ggpubr package in R software version 3.5.1/R studio version 1.1.456.

## 3. Results

### 3.1. Characterization of TiO_2_-NP Formulation

**Figure 1:**
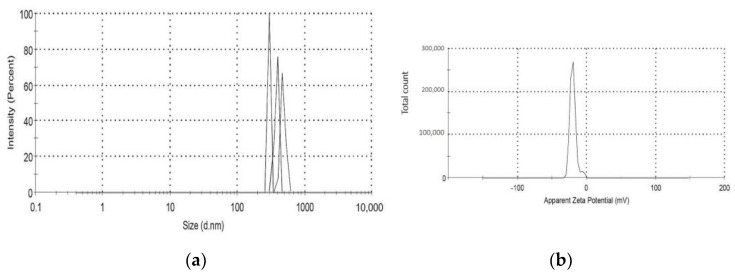
(**a**,**b**) shows the mean particle size and zeta potential of TiO_2_-NP.

**Figure 2:**
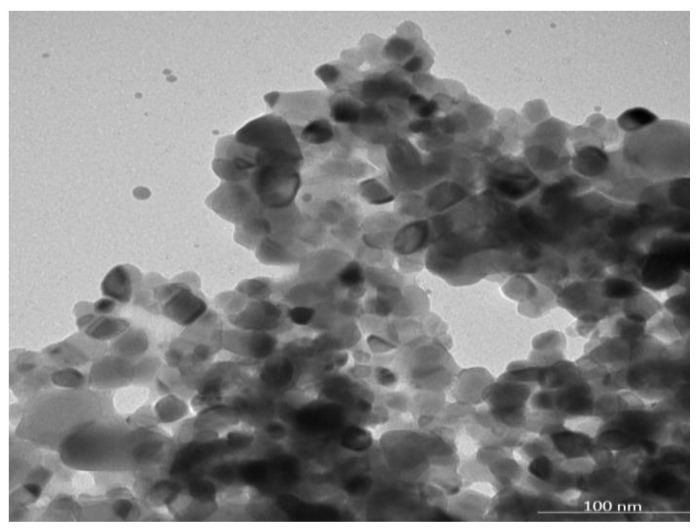
The morphology of the TiO_2_-NP by using transmission electron microscopy (TEM).

The Malvern Zetasizer determined that the particle size of the TiO_2_-NP formulation was 383.1 ± 23.05 nm (PDI = 0.931) and the negative zeta potential was 19.27 ± 4.65 mV (Figure 1). The transmission electron microscope (TEM) revealed the agglomeration of two distinct morphologies of particles, including spherical and polygonal shapes, with particle sizes ranging from 50 to 100 nm (Figure 2).

### 3.2. Antimicrobial and Antibiofilm-Forming Activities

Table 4 demonstrates that the TiO_2_-NP formulation exhibits antibacterial properties against *S. aureus* ATCC 25923 and *E. coli* ATCC 25922. Considering the Gram stain classification, Gram-negative bacteria appear to be more susceptible to almost all treatments, except positive control group. Gentamycin was the most effective agent against both microorganisms tested, whereas there were no statistically significant differences in zones of inhibition between TiO_2_-NP formulation, Ag-NP formulation (Nano Care: Silver nano formulation, Bangkok, Thailand), and Iodine solution (Betadine, Bangkok, Thailand) against *S. aureus*. Ag-NP formulation has high efficacy against *E. coli*.

**Figure 3:**
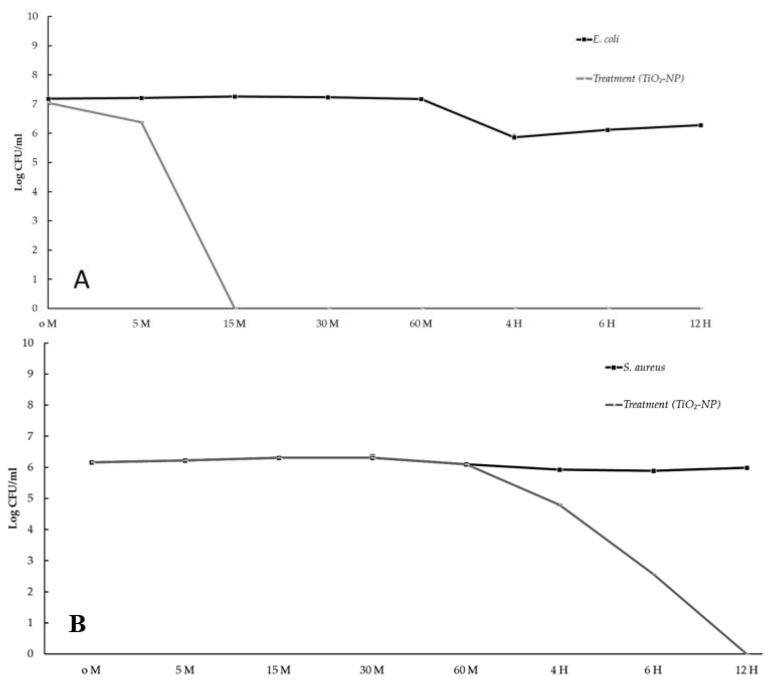
The time kill kinetic profiles of TiO_2_-NP formulation against *E. coli* (**A**) and *S. aureus* (**B**).

Bacteriostatic and bactericidal effects were evaluated using time-kill assays. Bacterial reductions of three logs of CFU/mL or greater from the initial inoculation concentration are considered bactericidal [28]. The results show that the TiO_2_-NP formulation had a bactericidal effect, lowering the starting log CFU/mL by more than 3 logs after 10 min against *E. coli* and after 6 h against *S. aureus*. No measurable growth of *E. coli* was observed 15 min after inoculation and lasted for 12 h, whereas no measurable growth of *S. aureus* was observed at the 12-h time point (Figure 3A,B).

**Figure 4:**
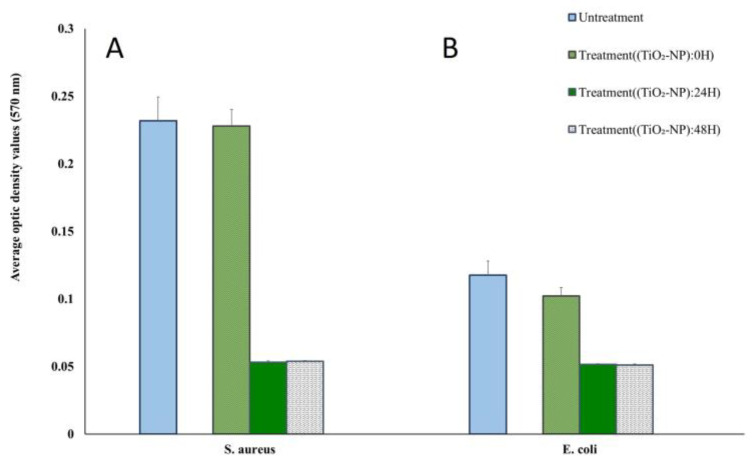
Biofilm formation ability of *S. aureus* (**A**) and *E. coli* (**B**) at different times of TiO_2_-NP formulation exposure compared with the untreated group.

The biofilm-forming ability was measured using the crystal violet method. All isolates tested were biofilm producers. Among the two reference bacterial strains, *S. aureus* was classified as a strong biofilm former (OD > 4OD_NC_), whereas *E. coli* was classified as a weak-producing biofilm former (OD_NC_ < OD ≤ 2 OD_NC_). The TiO_2_-NP formulation acted as an antibiofilm agent in all reference strains causing differences in biofilm formation and lower inhibition of initial cell adhesion compared to the negative control. The treatment of *S. aureus* and *E. coli* with TiO_2_-NP formulation for 24 h reduced biofilm formation by more than 99% (Figure 4).

### 3.3. Wound Healing Assay

#### 3.3.1. In Vitro

**Figure 5:**
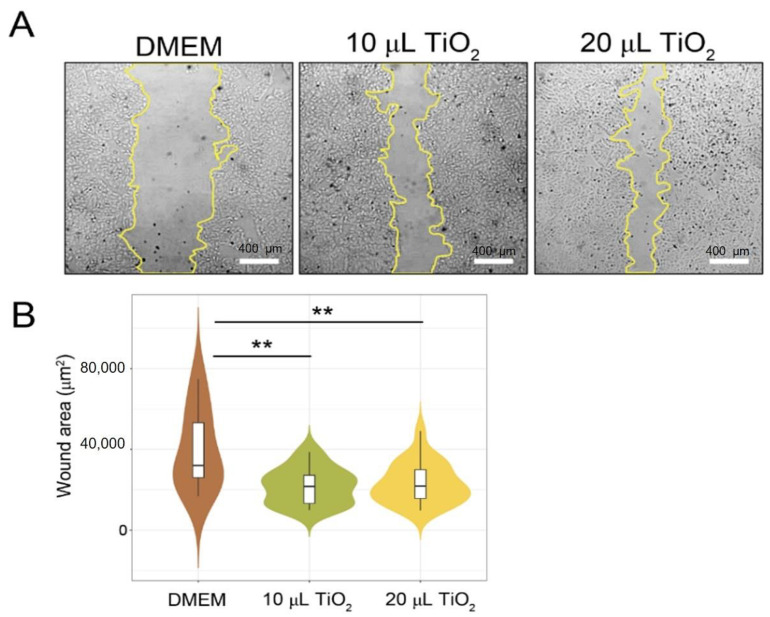
In vitro scratch wound healing assays demonstrating that cell migration into the cell-free region (**A**) is significantly different between the 10 and 20 µL as compared to the negative controls (DMEM). (**B**) A summary bar graph illustrating the percentage of wound closure at the indicated time points during the scratch wound. assay (** *p* < 0.01 compare with control).

According to the findings, the TiO_2_-NP formulation has an effect on the growth of swine kidney cells (PK15) in the in vitro scratch wound healing assay. A representation of both concentrations of TiO_2_-NP formulation significantly enhanced the proliferation and migration of the cells compared to the untreated control cells in a dose-dependent manner (*p* = 0.0016). Figure 5 shows that without TiO_2_-NP formulation supplementation, wound edges widened in comparison to those with TiO_2_-NP formulation supplementation at 10 µL and 20 µL. In comparison to the untreated control (32,033 µm^2^), TiO_2_-NP supplementation at 10 µL and 20 µL resulted in a median wound area of 21,646 µm^2^ and 21,887 µm^2^, respectively.

#### 3.3.2. In Vivo (Animal Model)

**Figure 6:**
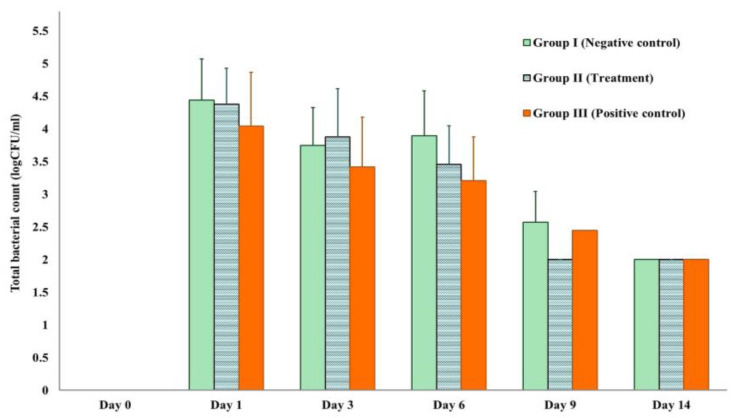
Total bacterial count measured by log CFU/mL in each experiment.

Each experiment was conducted on surgical wounds infected with *S. aureus*. Figure 6 illustrates the number of bacteria in CFU/mL for each treatment group with different durations, including the negative control (Group I: 0.85% NSS), the treatment (Group II: TiO_2_-NP formulation), and the positive control (Group III: Iodine solution), from Day 1 to Day 14. The results indicate that there is no difference in the number of bacteria between groups (*p* > 0.05). Nonetheless, it was found that the bacterial load decreased in all groups from the time of exposure.

The macroscopic evaluation of the wound did not show evidence of any purulent exudate in any of the wounded animals during study period. However, it was possible to observe mild inflammatory signs with a serous exudation, characterized by redness, and swelling at the edges of the wound on Day 0. In all groups, the wound healing exhibited a reduction in clinical signs after the surgery, as well as an increase in the rate of wound contraction and a decrease in wound area. To evaluate the physical appearance, we used the early wound healing score (EHS) and carried out semi-quantitative scoring comparisons between groups on different observation days.

**Figure 7:**
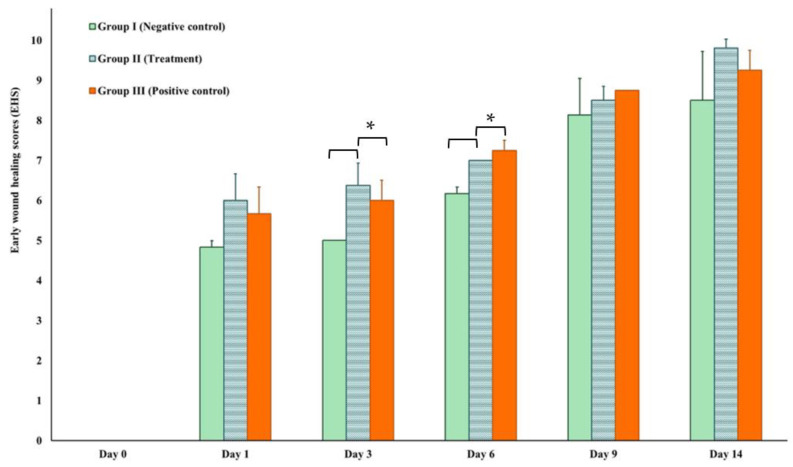
Early wound healing score (EHS) on Day 1, 3, 6, 9, and 14 compared to Day 0. * Asterisks indicate significant differences (*p* < 0.05).

The EHS was determined for the wounds in the early stage of healing, starting at 24 h after surgical approach and inoculation with *S. aureus* infection (Figure 7). The mean EHS values of the negative control, treatment, and positive control groups were 4.83 ± 0.167, 6.00 ± 0.67, and 5.67 ± 0.67, respectively. On Day 14, the treatment groups exhibited the highest EHS score of 9.81 ± 0.21, followed by the positive control group with a score of 9.25 ± 0.50 and the negative control group scoring 8.50 ± 1.22. On days 3 and 6, the treatment group showed statistically significant better scores (6.38 ± 0.96 and 7.00 ± 0.00) in comparison to the negative control groups (5.00 ± 0.00 and 6.17 ± 0.39) of *p* = 0.00128 and *p* = 0.000001, respectively.

**Figure 8:**
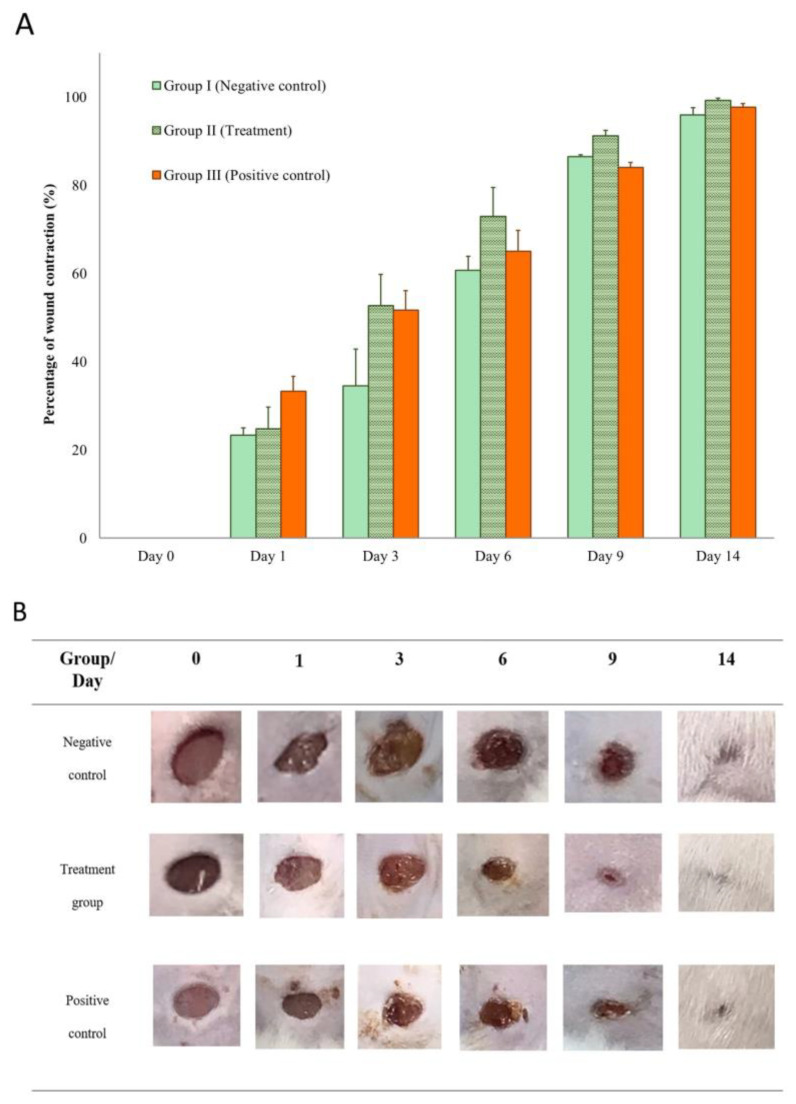
(**A**,**B**) Wound appearance and contraction on Days 0, 1, 3, 6, 9, and 14 showing comparison between groups (Group I: negative control (0.85% NSS), Group II: treatment (TiO_2_-NP formulation), and Group III: positive control (Iodine solution).

The percentages of the wound contraction induced by topical application of the TiO_2_-NP formulation, 0.85% NSS and iodine solution are shown in Figure 8A. Results demonstrate that treatment of wounds with the TiO_2_-NP formulation induced a significant (*p* < 0.05) improvement in the percentage of wound contraction (91.25 ± 2.50) compared to the negative control group treated with 0.85% NSS (86.50 ± 0.71). Starting on the third day of treatment, the TiO_2_-NP formulation showed significant wound contraction compared to the negative control group. The TiO_2_-NP formulation also showed better wound contraction than iodine solution from three to fourteen days after wounding, although the difference was not statistically significant.

The microscopic evaluation of the wounds was carried out by taking a standardized biopsy for histopathological analysis which encompassed the whole wound, including the edges. Hematoxylin and eosin (H&E), a commonly used staining method for dermatopathology, was employed for the descriptive analysis of wound healing parameters, including the inflammatory infiltrate, tissue granulation, primary fibrous scarring, and the epithelialization process. A comparative analysis was performed between the groups on days 0 and 14. The sectioning of the skin and subcutaneous samples obtained after surgical intervention and treatment in all groups revealed multifocal accumulation of the inflammatory cells predominantly composed of non-degenerated neutrophils, with fewer degenerated neutrophils, lymphocytes, macrophages, and foci of necrosis.

**Figure 9:**
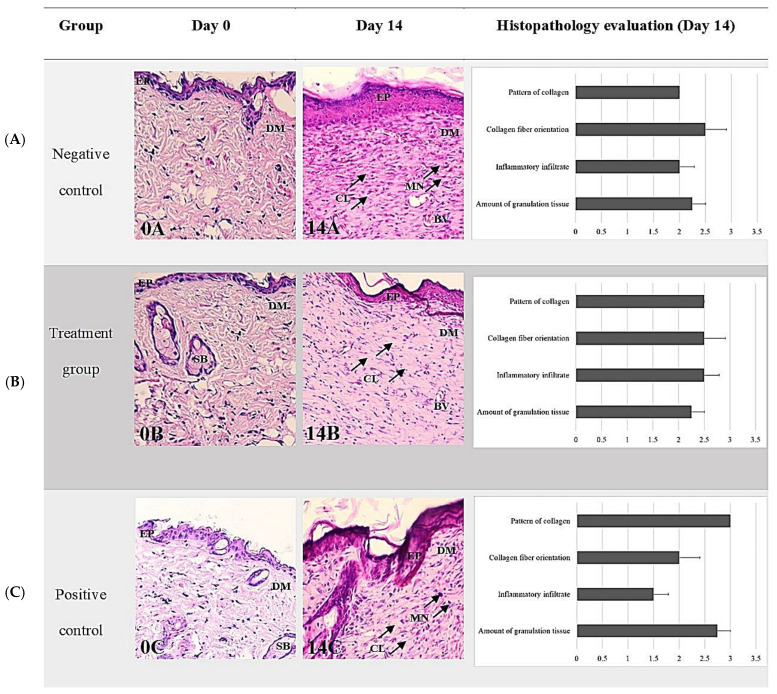
Representative histopathological sections stained with H&E of wound healing on Days 0 and 14 showing comparisons between groups ((**A**) negative control group; (**B**) treatment group (TiO_2_-NP formulation); (**C**) positive control group). Note the thin epidermis and dermis above the abundant subcutaneous layer on Day 0 (**A**–**C**) compared with Day 14 (**A**–**C**). The H&E staining shows collagen fibers—stained pale pink, cytoplasm stained purple, nuclei stained blue, and red blood cells—stained cherry red. The negative control had the thickest layer of epithelium compared to the other groups and showed remarkable vertical collagen formation (14A). The TiO_2_ NP formulation was randomly infiltrated with mononuclear cells (MN) and mainly vertical collagen formation (14B). The positive control demonstrates a mixed pattern of collagen fiber formation, infiltration of a moderate number of mononuclear cells (MN), and granulation tissue (14C). The wound exhibits a few to moderate numbers of infiltrating inflammatory cells, the majority of which are neutrophils, and a small number of macrophages and lymphocytes with foci of necrosis, ulceration, and fibrin formation (0A, 0B, 0C). Abbreviations: H&E, Hematoxylin-Eosin; EP, Epidermis, DM; Dermis, SB; Sebaceous gland, BV; Blood vessel, MN; Mononuclear cell infiltration (arrowhead), CL; Collagen fiber orientation (arrowhead).

The comparison of histological scoring between Day 0 and Day 14 was performed by three examiners who were blinded to the group information during all histological analyses. The parameters evaluated included the amount of granulation tissue, pattern of collagen, inflammatory infiltrate, and collagen fiber orientation. On Day 14, the treatment group that received TiO_2_-NP formulation showed a higher score for the amount of granulation tissue (2.75 ± 0.25) compared to the negative and positive control groups (2.25 ± 0.25, 2.25 ± 0.48). Additionally, the treatment group also showed the highest score for the pattern of collagen (3.00 ± 0.00) with remarkable vertical collagen formation. However, the inflammatory infiltrate and collagen fiber orientation were lower in the treatment group. In the positive control group, which was exposed with Iodine solution, the histological analysis revealed a mixed pattern of collagen fiber formation, infiltration of a moderate number of mononuclear cells (MN) and the presence of foci of ulceration and fibrin formation mixed with increasing granulation tissue. On Day 14, there was a statistically significant difference between treatment and control group as regards the pattern of collagen as assessed using the Kruskal–Wallis test (*p* = 0.045). The pairwise Dunn’s test between groups showed that only the difference between the negative control group (0.85% NSS) and treatment group (TiO_2_-NP) was significant (*p* = 0.0417). However, there was no significant difference between collagen parameters, including number and pattern of collagen fibers among any groups on Day 0 and Day 14 (Figure 9).

## 4. Discussion

Due to particle aggregation, the average particle size of the TiO_2_-NP formulation, as determined by DLS, was found to be larger than that observed by TEM. This is because the formulation’s zeta potential is less than +30 or −30 mV, resulting in greater incipient stability and a lower potential force to prevent particle aggregation and flocculation [29].

TiO_2_-NP is an interesting antimicrobial agent due to its outstanding antibacterial capabilities against a wide spectrum of Gram-positive and Gram-negative bacteria. Electrostatic interaction, oxidative stress caused by the generation of reactive oxygen species (ROS), and disruption of protein functions and cell structures caused by metal cation release, leads to cell death [30]. According to the findings, the TiO_2_-NP formulation is more effective against *E. coli* ATCC 25922 than *S. aureus* ATCC 25923. The observed phenomenon may be attributed to the variation in thickness of bacterial cell walls, which ranges from 20–80 nm in Gram-positive and 1.5–10 nm in Gram-negative bacteria [31]. Furthermore, previous research has shown that *E. coli* cell walls contain lipopolysaccharide, phosphatidyl-ethanolamine, and peptidoglycan and are susceptible to peroxidation induced by TiO_2_. Consequently, it is anticipated that the impact of TiO_2_-NP will exhibit slight variations depending on the type of microorganism involved [32].

The time-kill kinetic assay revealed similar antibacterial results. Specifically after 10 min and 6 h, the TiO_2_-NP formulation reduced *E. coli* and *S. aureus* by more than 3 logs. Pulgarin et al. [33] have reported comparable findings regarding the effectiveness of TiO_2_ thin film coated on the PVC substrate against *E. coli*. The bacterial count was observed to decrease from 6.90 × 10^9^ to 5.00 × 10^2^ CFU/mL within 60 min, indicating that over 99% of the *E. coli* population was eliminated. Furthermore, Di Pilato’s study [34] demonstrated that the time kill kinetics of *Staphylococcus* group organisms with significant biofilm formation, such as *S. aureus* and *S. epidermidis*, increased with exposure duration. Biofilm formation is a complex process that involves the attachment of microorganisms to surfaces and the production of an extracellular matrix. The matrix provides protection against environmental stressors such as antibiotics and host immune responses. *S. aureus* and *S. epidermidis* are two organisms that are known to form biofilms, and their ability to do so increases with exposure duration. This is concerning because biofilm formation is a significant factor in the increase in antimicrobial resistant bacteria. In fact, the biofilm produced by *S. aureus* may be the cause of increased antimicrobial resistance in wound sites [35], which can lead to serious infections that are difficult to treat. Therefore, understanding the mechanisms of biofilm formation and finding new ways to disrupt it may be key in combating antimicrobial resistance and improving patient outcomes.

We found that the TiO_2_-NP formulation acted as an antibiofilm agent in all reference strains. Similar to the findings published by Carrouel and Viennot, who observed that adding metal oxide, specifically TiO_2_, to toothpastes and mouthwashes increased their antibacterial effectiveness against dental-plaque-causing microbes [36]. Furthermore, Jesline et al. [13] demonstrated that TiO_2_-NP were effective against methicillin-resistant *Staphylococcus aureus* (MRSA) and *E. coli* by reducing biofilm formation by 40–50% [37]. TiO_2_-NP formulation can reduce the adhesion of bacteria and inhibit biofilms, primarily due to the generation of ROS and lipid oxidation on the cell wall membrane which leads to the destruction of bacteria inside the biofilm. In addition, the interaction between TiO_2_-NP and biofilm is determined by their electrostatic characteristics. The positive charge of the bacterial biofilm matrix can interact with negatively charged metal ions and organic compounds through electrostatic forces. Some studies have recently revealed that TiO_2_-NP has promising cell-growth-promoting characteristics in the wound healing process [38,39].

The present study also used an animal model to assess the in vivo efficacy of a TiO_2_-NP formulation for the treatment of *S. aureus*-infected wounds. The wounds in all groups showed only mild inflammation with serous exudation at the wound edges, which is a normal part of the healing process. At the time of exposure, the bacterial load decreased in all groups, and there is no difference in the number of bacteria between groups (*p* > 0.05) that shown in Figure 6. This can be explained due to all healthy mice used in this study having a normal defense mechanism to eliminate pathogens and reduce the bacterial load to the normal level of commensal organisms on the skin. These findings add weight to previous studies that found the health and immune status of a patient have a significant impact on the bacterial diversity and population of the cutaneous microbiota [40].

The endpoint of a successful treatment is complete and permanent wound closure. Our studies have shown that the TiO_2_-NP formulation was able to enhance cell proliferation and migration (scratching wound healing assay), which is crucial to wound healing, especially in the inflammatory reaction stage. After the inflammatory response, inflammatory cells send chemotactic signals to stimulate fibroblast migration and start granulation tissue formation. The formation of granulation tissue can typically be observed within 3–5 days after the injury, and our results have shown that the group treated with TiO_2_-NP formulation had a higher EHS score during the process of formation of the granulation tissue (Figure 7). In addition, abundant extracellular matrix has accumulated in the myofibroblast phenotype, which is associated with a statistically significant difference in the percentage of wound contraction between the groups.

Histological analysis of the group receiving the TiO_2_-NP formulation had significantly higher scores for granulation tissue and collagen patterns groups at the end of the study. The arrangement and orientation of collagen plays a crucial role during the remodeling phase, which influences the appearance of the final scar after wound closure. The group treated with TiO_2_-NP formulation exhibited a vertical collagen orientation, whereas the iodine solution group displayed a mixed pattern of collagen fiber formation, suggesting that the main difference between scarred and unwounded skin appears to be the density, uniformity, size, and orientation of the collagen fibrils. The presence of a mixed pattern of collagen fibers in the iodine solution group is characterized by non-uniformity and low density, which can potentially contribute to the appearance of unhealed skin [41]. In this study, the inability to determine the types of collagen fibers is a limitation of using H&E staining to evaluate wound healing. In prior studies, additional techniques were utilized to determine the presence of type I and type III collagen in wound healing sites. These techniques include Herovici’s polychrome staining with multispectral imaging [42] and a combination of histochemical staining and immunostaining on normal and injured skin samples at various stages of healing [43]. The histological analysis of the healed wound tissues on day 14 was consistent with the visual observation and grading. This suggests that the TiO_2_-NP formulation is an acceptable wound healing agent, with outcomes comparable to the positive control group, and superior to the negative control group.

## 5. Conclusions

TiO_2_-NP formulation is a highly effective antimicrobial and antibiofilm agent. These properties make it a promising candidate for use in medical applications, particularly with regard to wound healing. However, formulation instability caused by particle aggregation should be reduced. With further research and development, TiO_2_-NP could become a valuable tool in the fight against bacterial infections and in promoting tissue regeneration. Its unique properties make it a compelling alternative to traditional antibiotics and wound care treatments.

## Figures and Tables

**Table 1:** Classification of biofilm formation abilities.

Cut-Off Value Calculation	Biofilm-Formation Abilities
OD ≤ OD_NC_	Non-biofilm-forming
OD_NC_ < OD < 2OD_NC_	Weak-biofilm-forming
2OD_NC_ < OD < 4OD_NC_	Moderate-biofilm-forming
4OD_NC_ < OD	Strong-biofilm-forming

Abbreviations: OD_NC_: OD of negative control group, OD: OD of treatment group.

**Table 2:** Early wound healing score descriptions.

Parameter	Descriptions	Score
CSR	Merged surgical wound margins	6
	Surgical wound margins in contact	3
	Visible distance between surgical wound margins	0
CSH	Absence of fibrin on the surgical wound margins	2
	Presence of fibrin on the surgical wound margins	1
	Bleeding at the surgical wound margins	0
CSI	Absence of redness along the surgical wound diameter	2
	Redness involve < 50% of the diameter	1
	Redness involve > 50% of the diameter and/or pronounced swelling	0

Abbreviations: CSR: clinical signs of re-epithelialization, CSH: clinical signs of hemostasis, CSI: clinical signs of inflammation.

**Table 3:** Histological parameters used to assess and calculate wound healing state.

Histological Parameters	Scoring System	Histological Grading
Amount of granulation tissue	Profound	1
	Moderate	2
	Scanty	3
	Absent	4
Inflammatory infiltrate	Plenty	1
	Moderate	2
	A few	4
Collagen fiber orientation	Vertical	1
	Mixed	2
	Horizontal	4
Pattern of collagen	Reticular	1
	Mixed	2
	Fascicle	4

**Table 4:** Zones of inhibition of metal and metal-oxide nano formulations against reference bacterial strains.

Bacteria		Inhibition Zones (mm)			
	N	TiO_2_-NP	Ag-NP	Iodine Solution	Gentamycin
*S. aureus*	3	20.00 ± 5.42	20.12 ± 0.18	22.23 ± 0.24	31.75 ± 0.37 *
*E. coli*	3	24.00 ± 1.91	29.50 ± 0.50 *	24.51 ± 0.43	30.50 ± 0.75 *

* Asterisks indicate significant differences (*p* < 0.05).

## Data Availability

The data presented in this study are available upon request from the corresponding author. The data are not publicly available due to the privacy policy of the authors’ institution.
